# Biocatalytic recycling of plastics: facts and fiction

**DOI:** 10.1039/d5sc00083a

**Published:** 2025-03-31

**Authors:** Wolfgang Zimmermann

**Affiliations:** a Leipzig University, Institute of Analytical Chemistry Johannisallee 29 04103 Leipzig Germany wolfgang.zimmermann@uni-leipzig.de

## Abstract

Due to the lack of efficient end-of-life management, the mass production of plastics has resulted in serious environmental problems. Sustainable biological approaches using enzymes to degrade and recycle plastic waste are emerging as a complement to conventional methods to promote a circular economy of plastics. Only a fraction of the plastic waste generated is currently suitable for biocatalytic deconstruction and the development of economically and environmentally competitive processes is still pending. Inconsistent claims about new plastic-degrading enzymes reveal a need for robust and standardized analysis methods to ensure reproducible results and a realistic evaluation of their potential. This paper critically reviews enzymatic synthetic polymer degradation and its recycling challenges.

## Abstract 2

Enzymatic recycling of plastic waste is attracting considerable attention as a novel strategy to advance a circular plastic economy. While currently limited to polyesters, achieving economically viable biocatalytic processes remains a key challenge.

## Graphical abstract

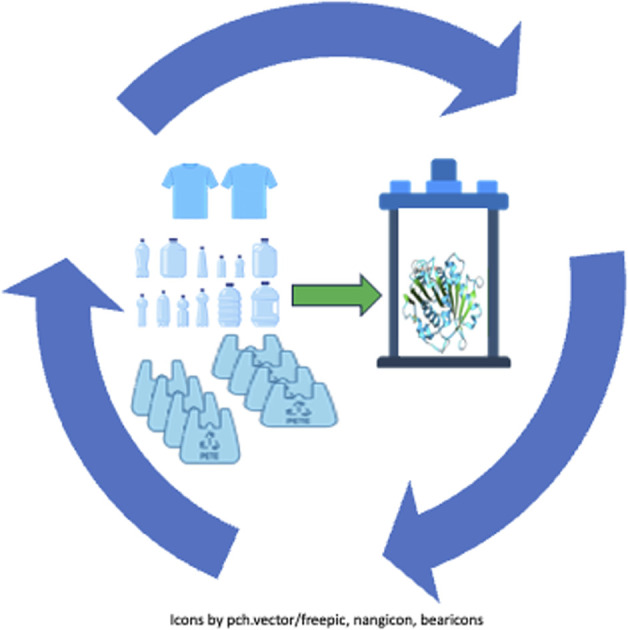

## Introduction

Since the 1950s, billions of tons of plastics made from a plethora of polymers have been produced from fossil feedstocks powered by the quest of the petrochemical industries to find proliferating markets alongside growing consumer demands for mass-produced versatile and affordable commodities. The level of global plastics pollution resulting from their overabundance is increasingly perturbing essential Earth system processes.^1^ The massive greenhouse gas emissions from the production of plastics are contributing to climate change and have been estimated to reach without intervention 15% of the global carbon budget by 2050.^2^ The vast majority of the waste generated from plastic products ends up in landfills, is incinerated or is released into the environment, and only a fraction is recycled for further use.^3^ For many years, this systematic mismanagement of plastic waste has not posed a major concern for plastic producers, governments, or even the consumers. However, the ever-growing pollution of land, rivers, lakes, and oceans with plastics and the ensuing formation of micro- and nano-plastics has increased awareness of a major threat to our ecosystems and to human health.^4,5^

Efforts driven by more stringent legislative mandates and consumer demands to support a circular economy of plastics are aiming to substantially increase the recycling rates of plastic wastes and to extend the lifetime of plastic products. In this context, biocatalytic approaches for plastic recycling have developed into a burgeoning area of research.^6–9^ The biocatalytic recycling of polyesters has reached industrial scale with the potential to become a relevant complementary technology for plastic waste management.

Microorganisms and biocatalysts reported to be able to degrade plastics have received considerable attention in the scientific community and media, with news outlets portraying “plastic-eating” microbes and enzymes as a solution for the global plastic crisis. A closer look at some of the reports on plastic-degrading enzymes suggests that claims of new results and achievements should not be overestimated to prevent public and peer wrong expectations and misconceptions about enzymatic plastic degradation and recycling.

### Plastic waste recycling

**Fig. 1:**
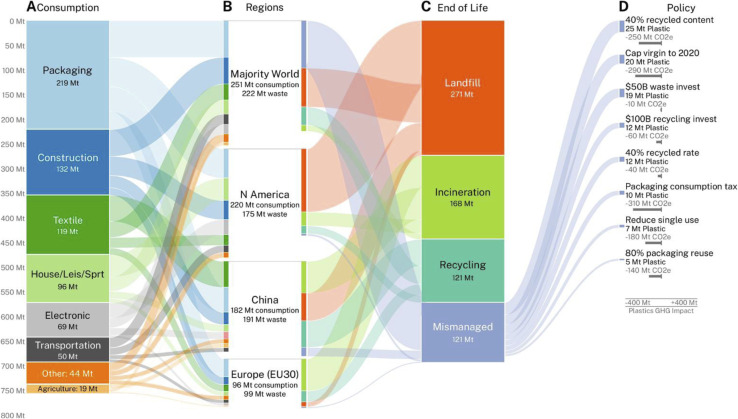
Global plastics projections. Overall mass of plastics (million metric tons) predicted in 2050 to (A) be consumed in eight global sectors, (B) in four world regions and (C) in four end-of-life fates. (D) Estimated impact of policy interventions on reducing mass of mismanaged plastic waste and greenhouse gas emissions (million metric tons CO_2_e) in 2050 (reproduced with permission from ref. 10).

The global annual volume of plastic waste generated has been estimated to reach without intervention a staggering 700 million metric tons in 2050 (ref. 10) (Fig. 1). Plastic waste often contains several types of synthetic polymers in complex mixtures, additives and contaminants complicating their potential recycling.^11,12^ Single-use plastics, for example bags, bottles, and food packaging to be used once and then discarded represent a large share on the total plastic waste produced. Polyolefins (polyethylene, PE, and polypropylene, PP) and polyvinyl chloride (PVC) are the main types of plastic waste and represented almost 58% of the global plastics production of 400 million metric tons in 2022 while polyesters such as polyethylene terephthalate (PET) and polyurethanes (PU) contributed about 6.2% and 5.2%, respectively.^13^

To mitigate the impact of accumulating global plastic waste, the development of advanced recycling technologies is one of the key strategies to increase the reuse of plastics and to diminish the demand for petrochemical feedstocks.^14^ Plastic waste recycling is mainly performed by thermo-mechanical or chemical methods.^15,16^ For the thermo-mechanical recycling of polyesters, for example post-consumer PET bottles, the sorted plastic waste is melted and extruded using high temperatures. The secondary materials produced from the recycled PET are typically of reduced quality and economic value. While the majority of plastic recycling is performed with this technology, the final disposal of the plastic waste is only postponed and hardly affecting virgin plastic production volumes from petrochemicals.^17,18^ By chemical recycling of PET, for example by glycolysis or methanolysis methods, the polymer is converted to its building blocks which can be used for the re-synthesis of virgin-grade plastics enabling a closed-loop recycling.^19,20^ Significant innovation and expanded PET recycling capacities using chemical methods can be expected in the future. For example, a PET recycling plant using a low-temperature glycolysis process with a small environmental impact and a production capacity of 280 metric tons recycled PET per day is planned to be in operation in India in 2025.^21^

### Enzymatic recycling of different plastic waste streams

Plastics exhibit a vast diversity of chemical structures with different susceptibility to biological degradation. The recalcitrant properties of PE, PP and PVC due to the lack of functional groups in their homochain polymer backbone requiring carbon–carbon bond cleavage and dehalogenation reactions provide these hydrophobic synthetic polymers with a high resistance to enzymatic degradation.^22^ In contrast, heterochain polyesters such as PET and PU contain hydrolysable bonds enabling an enzymatic deconstruction. A growing number of enzymes with activity against PET has been described, some of them suitable for applications in industrially relevant polyester recycling processes.^7,9,23^ Similar to chemical recycling, PET materials are deconstructed in a biocatalytic process to produce the constituent monomers which are recovered and used for the manufacture of new polyester comparable to PET made from petrochemicals.^24–27^ Different from chemical recycling methods, the biocatalytic process is performed with high selectivity under mild reaction conditions avoiding high temperatures and pressure. For example, the chemical depolymerization of PET by glycolysis, a transesterification reaction between PET and ethylene glycol, may require reaction temperatures of 160–300 °C and ≥1.1 MPa pressure.^14^

Post-consumer PET has been the main plastic waste targeted for biocatalytic recycling.^6,28^ The recycling of post-consumer PET bottles and fibres has been scaled up and a processing plant using engineered polyester hydrolases with a capacity of 50.000 metric tons per year is scheduled to be commissioned in France in 2026.^29^

### The predominant types of plastic waste are not efficiently deconstructed by enzymes

With 105 and 76 million metric tons produced globally in 2022, respectively, the polyolefins PE and PP significantly contribute to global plastic waste generation.^13^ PE is widely used for packaging, construction, automotive and industrial applications. Flexible, low-density PE is found in insulations, plastic bags, and food packaging while rigid high-density PE is used *inter alia* in detergent bottles and water pipes. PP has a wide range of applications in plastic products with high strength and stiffness and displays an even higher chemical resistance and biological recalcitrance compared to PE. Although a large number of publications have reported biodegrading effects of microorganisms on polyolefins, only limited information is available on potential enzymes involved and their mode of action.^30–32^ Most studies estimated degradation effects based on weight losses, polymer surface modifications or Fourier-transform infrared (FTIR) spectroscopic analysis while few reports used methods such as carbon isotope labelling^33^ to unequivocally prove and quantify a microbial mineralization by microorganisms. Polyolefin samples used as substrates for enzymes are often pre-treated by UV-irradiation or oxidizing agents to introduce functional groups and to achieve an initial reduction in the chain length of the polymer to promote their enzymatic degradation.^34,35^ The degradation effects observed following incubation with enzymes could thereby be limited to the shorter segments of the PE chains created by the abiotic pre-treatment or even originated from the degradation of non-polymeric additives often present in commercial polymer samples.^36^ Most studies used branched PE of low molecular weight and crystallinity as enzyme substrates which is more susceptible to an enzymatic attack. Typically, slow degradation rates, changes in the molecular mass distribution and modifications of the surface of PE films are observed by treatment with microbial redox enzymes such as multicopper oxidases including laccases and peroxidases for the introduction of functional groups and polymer chain cleavages.^37^ However, conclusive evidence for significant degradation of unadulterated high-molecular-weight polymer samples by these enzymes, and their biochemical mechanism, is lacking.^38^ The degradation of PE to low molecular weight products for recycling following the initial introduction of functional groups and chain cleavages will require additional enzymes such as alcohol dehydrogenases, monooxygenases, and hydrolases which complicates the enzymatic deconstruction process.^37^ A combination of chemical and enzymatic methods might provide a more viable strategy for biological polyolefin recycling. For example, a chemically pre-treated low-molecular weight PE was partially degraded to medium-sized carboxylic acids after incubation with an enzyme cascade composed of a catalase-peroxidase, an alcohol dehydrogenase, a Baeyer–Villiger monooxygenase, and a lipase.^39^

The homochain polymer PVC with a C–C backbone is resistant to many chemicals and highly recalcitrant to biological degradation. In 2022, it was the third-most widely produced plastic with about 51 million metric tons (ref. 13) and is applied in construction, automotive, and consumer goods. While certain degradation effects of mostly pre-treated PVC materials by various fungi and bacteria have been observed,^40–43^ convincing evidence for a substantial enzymatic degradation of PVC has not been demonstrated yet. PVC contains a high proportion of plasticizers potentially susceptible to microbial degradation which could result in an overestimation of an observed polymer degradation. A degradation of a PVC sample by a bacterial catalase-peroxidase indicated by FTIR spectroscopy and size exclusion chromatography (SEC) has been reported.^44^ In a follow-up study, the detected changes in molecular mass distribution following the incubation of PVC with the enzyme could not be confirmed. A surface oxidation of the polymer was also not validated by FTIR spectroscopy suggesting that the FTIR spectroscopic analysis of the PVC sample by Zhang *et al.*^44^ was influenced by protein adsorption on the polymer surface.^45^

None of the previously reported enzymes show sufficient activities against polyolefins and PVC which could make them suitable for applications in plastic recycling processes on any larger scale.

### Hydrolases degrading synthetic polyesters are promiscuous enzymes unlikely to have evolved since the 1950s

Polyester hydrolases have been detected in numerous fungi and bacteria, prevalently Actinomycetota from soil and compost habitats containing decaying plant materials where they play a crucial role in the recycling of plant biomass.^46^ Among a wide range of plant polymer-degrading enzymes, many thermotolerant and thermophilic Actinomycetota produce ester-hydrolysing cutinases degrading the plant polyesters cutin and suberin.^47^ Actinomycetota have become an import source of thermostable synthetic polyester-hydrolysing enzymes and their cutinases have been served as scaffolds for the construction of engineered variants suitable for biocatalytic recycling processes.^7,9,48^ The ability of cutinases to degrade structurally similar synthetic polyesters such as PET is likely the result of the low substrate specificity of these naturally occurring hydrolases enabling the hydrolysis of a broad spectrum of aliphatic and aromatic polyesters.^49,50^

A mesophilic polyester hydrolase-producing bacterium capable of assimilating PET was isolated from environmental samples containing plastic waste.^51^ Following this report, it has been speculated that the ability of microorganisms and their enzymes to degrade synthetic plastics is a result of their exposure to plastics in the environment.^29,52–54^ However, a mineralisation of PET by other polyester hydrolase-producing bacteria, for example Actinomycetota, has not been demonstrated using robust methodology such as stable carbon isotope labelling. Furthermore, microorganisms with PET-hydrolysing enzymes have been detected universally in many natural environments and metagenomes including pristine regions devoid of plastic contaminations.^55–57^ It is therefore debatable whether polyester hydrolases have evolved to degrade synthetic plastics since the introduction of plastic waste into the environment in the 1950s.

### High-crystallinity PET is not deconstructed by polyester hydrolases

The thermoplastic polyester PET is a heterochain polymer composed of terephthalic acid and ethylene glycol. PET has numerous applications in the manufacture of bottles and food packaging as well as textile fibers and is worldwide the most recycled type of post-consumer plastics. The polymer is composed of amorphous and crystalline regions with distinct differences in susceptibility to enzymatic degradation. Amorphous and low-crystallinity PET waste, for example thermoform food packaging, is readily deconstructed by thermostable polyester hydrolases and their engineered variants.^24,26,58^ Important other PET waste streams such as beverage bottles and textile fibres composed of high-crystallinity PET are not directly hydrolysable by enzymes and require a high-temperature pre-treatment step to convert the crystalline PET to amorphous material amenable to enzymatic hydrolysis.^26,59–61^ The detection of hydrolysis products obtained by treatment of high-crystallinity PET samples with polyester hydrolases^62–66^ is not a definitive proof of their ability to degrade crystalline PET since the products could originate from the hydrolysis of non-crystalline parts of the sample.^7^

Large volumes of textile waste containing polyester fibres are generated which are mostly landfilled or incinerated causing growing environmental issues.^67,68^ The biocatalytic recycling of PET in post-consumer textiles has gained interest due to the tolerance of the enzymatic process to additives, colorants and other types of fibres in mixed textiles. However, like PET beverage bottles, this process necessitates a thermomechanical pre-treatment with a high energy demand for the amorphization of the high-crystallinity PET fibres.^69–71^

The recalcitrant properties of high-crystallinity PET make significant direct deconstruction in a recycling process impossible with previously reported enzymes.

### Highly active and stable polyester hydrolases are required in industrial biocatalytic PET recycling processes

The enzymatic hydrolysis of the amorphous parts of the semi-crystalline polyester PET is most effective at reaction temperatures ∼70 °C near its glass transition temperature (*T*_g_).^72,73^ This temperature offers a trade-off between polymer mobility required for enzyme accessibility and polymer aging occurring at higher temperatures decreasing enzyme efficiency.^60^ Mesophilic polyester hydrolases are able to partially hydrolyse amorphous PET at temperatures around 30 °C. Due to their low stability, they do not catalyse the degradation of the bulk polymer at reaction temperatures required for a substantial deconstruction of the polymer.^28^ The complete enzymatic hydrolysis of amorphous PET in a large-scale industrial process with high substrate loadings within short reaction times at ∼70 °C is requiring highly active biocatalysts with thermostable properties. Only a limited number of polyester hydrolases and their variants have been identified to perform adequately under these conditions.^23^

Dual enzyme systems composed of a polyester hydrolase and a carboxylesterase to facilitate the degradation of intermediate PET hydrolysis products^74–78^ are unlikely to be required for large-scale PET deconstruction, which can be efficiently achieved with single, highly active polyester hydrolases.^23^

### Potential for the enzymatic recycling of other types of polyester plastic waste

Thermoplastic or thermoset PU containing polyester, polyether, and urethane bonds are versatile materials with a wide range of applications in insulations, coatings, footwear, automotive and construction. PU is another major contributor to global plastic waste accumulation, despite recycling opportunities.^79^ Considering their prospects for a biocatalytic recycling of PU, polyester hydrolases are able to partially hydrolyse ester bonds in some types of PU materials. For a conversion of the polymer to low molecular weight compounds, further enzymes such as urethanases and amidases to cleave the different types of bonds in PU would be required in an efficient biocatalytic degradation process.^80^ The inherent structural diversity of PU waste may however necessitate a combination of chemical pretreatments and biocatalytic depolymerization to obtain high-value reusable compounds in innovative PU recycling strategies.^79^

Plastic wastes from polyesters such as poly(ethylene furanoate) (PEF) as a biobased alternative to PET, the biodegradable thermoplastic polyester polylactic acid (PLA), polybutylene adipate terephthalate (PBAT), and polybutylene succinate (PBS) which are efficiently degraded by polyester hydrolases could also provide feedstocks for future biocatalytic recycling processes.^7,81,82^ Due to the small market shares, their recovery has currently not a large impact on total plastic recycling rates.

### Biocatalytic PET recycling needs to become competitive

**Fig. 2:**
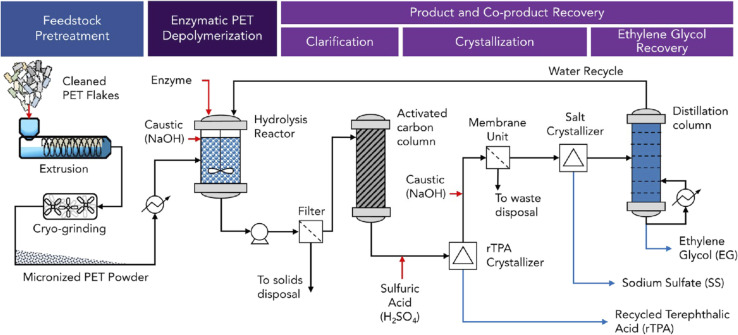
Schematic PET recycling process with polyester hydrolases. An amorphization and micronization pre-treatment is shown for the enzymatic recycling of high-crystallinity PET feedstock. The enzymatic hydrolysis is performed in a stirred-tank reactor with pH control using NaOH to neutralize released terephthalic acid (rTPA). The recovery of the hydrolysis products rTPA by crystallisation and of ethylene glycol (EG) by distillation in the downstream process is depicted. Red arrows indicate raw material inputs, and blue arrows product and co-product streams (reproduced with permission from ref. 92).

A process-based life cycle analysis indicated that an enzymatic recycling process using high-crystallinity post-consumer PET waste such as beverage bottles has a higher environmental impact compared to PET from petrochemical sources^83^ (Fig. 2). The thermomechanical melt extrusion treatment to convert high-crystallinity PET to amorphous materials suitable for enzymatic hydrolysis was identified as a major factor contributing to the high energy consumption and greenhouse gas emissions of the modelled process.^70^ A transformation to renewable energy sources for electricity generation and the development of low-energy pre-treatment methods could reduce greenhouse gas emissions and costs of enzymatic high-crystallinity PET recycling. Post-consumer clear PET beverage bottles for which cost-efficient mechanical recycling methods with lower environmental impact already exist may represent a less suitable waste stream for biocatalytic recycling.^84^

A minimum selling price of about USD 2 per kg for terephthalic acid produced with an enzymatic recycling process using high-crystallinity PET feedstock based on a post-consumer bale price of about USD 0.7 per kg has been estimated.^83^ Considering the continuing low market price for terephthalic acid and PET from petrochemical sources (USD 0.66 to USD 1.01 per kg for terephthalic acid and USD 0.67 to USD 1.52 per kg for PET in March 2025 (ref. 85 and 86)), it is evident that enzymatic recycling processes must significantly reduce costs to compete effectively with established market prices.

The price of post-consumer PET feedstock is strongly affecting the operating costs of a biocatalytic recycling plant.^87^ PET waste streams containing multilayer plastics or low-crystallinity thermoform PET packaging which do not require a high-energy pre-treatment for enzymatic deconstruction are typically mostly landfilled or incinerated. The bale prices for these post-consumer plastics are significantly lower compared to clear post-consumer PET bales from beverage bottles.^20^ These plastic waste streams have been shown suitable for enzymatic recycling and could be considered as alternative feedstocks for biocatalytic processes integrating into existing recycling schemes.^6,9^ However, inadequate plastic waste collection and inefficient separation presently significantly limit the recycling of these feedstocks in many countries. The extent of their utilisation will depend on the establishment of suitable waste collection infrastructure and the development of advanced sorting technologies.^88,89^

### Standardized plastic samples and appropriate analytical methods are important for accurate assessment of plastic-degrading enzyme activities

Results on enzymatic plastic-degrading activities obtained from different studies are often difficult to compare. In many reports, non-standardised and poorly characterised plastic samples were used as enzyme substrates, lacking essential details about their exact formulation, molecular mass, and crystallinity. To add to the complexity of the composition of plastic substrates used for enzymatic degradation studies, the presence of additives such as stabilizers, fillers and plasticizers common in commercial samples further complicates an interpretation and comparison of the results.^90^

The use of gravimetric analyses to demonstrate weight losses of the treated polymer materials can lead to an overestimation of enzymatic activity in the presence of easily degradable non-polymer compounds in the plastic sample.^91^ FTIR spectroscopy is often used to detect a biological oxidation of homochain polymer samples evidenced by the formation of C=O, C–O and O–H bonds. However, analyses based on FTIR can lead to ambiguous results due to misinterpretations of the spectra, for example in the presence of additives or contaminants in the sample.^92,93^ The oxidation and depolymerisation of PE powder by hexamerin proteins with phenoloxidase activity in the saliva of *Galleria mellonella* larvae has been claimed based on analysis by FTIR spectroscopy and SEC.^94^ In a later study, an enzymatic oxidation and degradation of additive-free PE films could not be confirmed by FTIR spectroscopic analysis and SEC indicating that the peaks in the FTIR spectra observed by Sanluis-Verdes *et al.*^94^ likely originated from protein bound to the film and not from an enzymatic modification of the PE sample.^45^

### Recycling alone will not solve the plastic crisis

Projections on global plastics based on current trends for production, consumption, and end-of-life management suggest that by 2050, without significant intervention, less than 20% of the total amount of globally produced plastic waste will be recycled, while about 120 million metric tons will contribute to mismanaged plastic pollution^10^ (Fig. 1). While supporting the transition to a circular economy of plastics, recycling alone is insufficient to address the plastic pollution crisis.

An essential step forward will be globally implemented mandates such as proposed in the Environment Programme of the United Nations to limit plastic production from petrochemical feedstocks.^95^ This demand is however continuously met with stiff opposition from a number of oil-producing countries. A model to forecast the impact of different policy interventions indicated that a 40% minimum recycled plastic content mandate and a cap to global virgin plastic production at 2020 levels are among the most effective measures to substantially reduce mismanaged plastic waste.^10^ The development of novel types of plastics designed for circularity represents another important strategy for reducing the accumulation of recalcitrant plastic waste.^96^ Implementing robust extended producer responsibility schemes and a policy-driven phasing out of single-use products supported by fostering public awareness to change consumer behavior constitute additional critical measures to significantly reduce global plastic pollution.^97,98^

## Conclusions

The potential of biocatalysis for synthetic polymer deconstruction and its application in plastic waste recycling processes has garnered high attention. While plastic degradation using enzymes offers an appealing green alternative to conventional plastic recycling methods, their potential to deconstruct important types of waste plastics is presently limited. A large-scale enzymatic recycling of polyolefins or PVC waste is currently not feasible. Reports on enzymes degrading these major plastic waste polymers must use rigorous analytical methods and thorough biochemical characterisation of the enzymes to obtain reliable data on their catalytic potential.

Biocatalytic PET deconstruction employing advanced polyester hydrolases with high performance and stability has emerged as an early-stage plastic recycling technology. The process costs and environmental impact of enzymatic recycling must be addressed to compete with advanced chemical and mechanical recycling methods. The implementation of decarbonized energy in the recycling process and the improvement of pre-treatment methods for important plastic waste streams like PET bottles and fibres will significantly enhance the cost-efficiency and environmental performance of biocatalytic recycling. The demand for recycled PET is expected to increase in response to regulatory recycled content targets creating opportunities for biocatalytic methods to process currently unutilised plastic waste streams. Increased plastic recycling capacity and binding global policies to limit petroleum-based plastic production will be essential for a circular economy of plastics and solving the global plastic crisis.

## Data availability

No primary research results, software or code have been included and no new data were generated or analysed as part of this review.

## Author contributions

WZ conceived and wrote the manuscript.

## Conflicts of interest

The author has filed the European patent application EP4455200A2 on enzyme-based degradation of plastics.
